# When randomization is not enough: divergent β-blocker trials and the illusion of universal causality

**DOI:** 10.1093/ehjcvp/pvag037

**Published:** 2026-06-01

**Authors:** Elmir Omerovic, Truls Råmunddal, Sandeep Jha, Petur Petursson, Kristofer Skoglund, Araz Rawshani, Björn Redfors

**Affiliations:** Department of Cardiology, Sahlgrenska University Hospital, 41345 Gothenburg, Sweden; Department of Molecular and Clinical Medicine, Institute of Medicine, Sahlgrenska Academy, University of Gothenburg, 41345 Gothenburg, Sweden; Department of Cardiology, Sahlgrenska University Hospital, 41345 Gothenburg, Sweden; Department of Molecular and Clinical Medicine, Institute of Medicine, Sahlgrenska Academy, University of Gothenburg, 41345 Gothenburg, Sweden; Department of Cardiology, Sahlgrenska University Hospital, 41345 Gothenburg, Sweden; Department of Molecular and Clinical Medicine, Institute of Medicine, Sahlgrenska Academy, University of Gothenburg, 41345 Gothenburg, Sweden; Department of Cardiology, Sahlgrenska University Hospital, 41345 Gothenburg, Sweden; Department of Molecular and Clinical Medicine, Institute of Medicine, Sahlgrenska Academy, University of Gothenburg, 41345 Gothenburg, Sweden; Department of Cardiology, Sahlgrenska University Hospital, 41345 Gothenburg, Sweden; Department of Molecular and Clinical Medicine, Institute of Medicine, Sahlgrenska Academy, University of Gothenburg, 41345 Gothenburg, Sweden; Department of Cardiology, Sahlgrenska University Hospital, 41345 Gothenburg, Sweden; Department of Molecular and Clinical Medicine, Institute of Medicine, Sahlgrenska Academy, University of Gothenburg, 41345 Gothenburg, Sweden; Department of Cardiology, Sahlgrenska University Hospital, 41345 Gothenburg, Sweden; Department of Molecular and Clinical Medicine, Institute of Medicine, Sahlgrenska Academy, University of Gothenburg, 41345 Gothenburg, Sweden

**Keywords:** causality, beta blockade, myocardial infarction, randomized controlled trials

## Abstract

**Structured graphical abstract:**
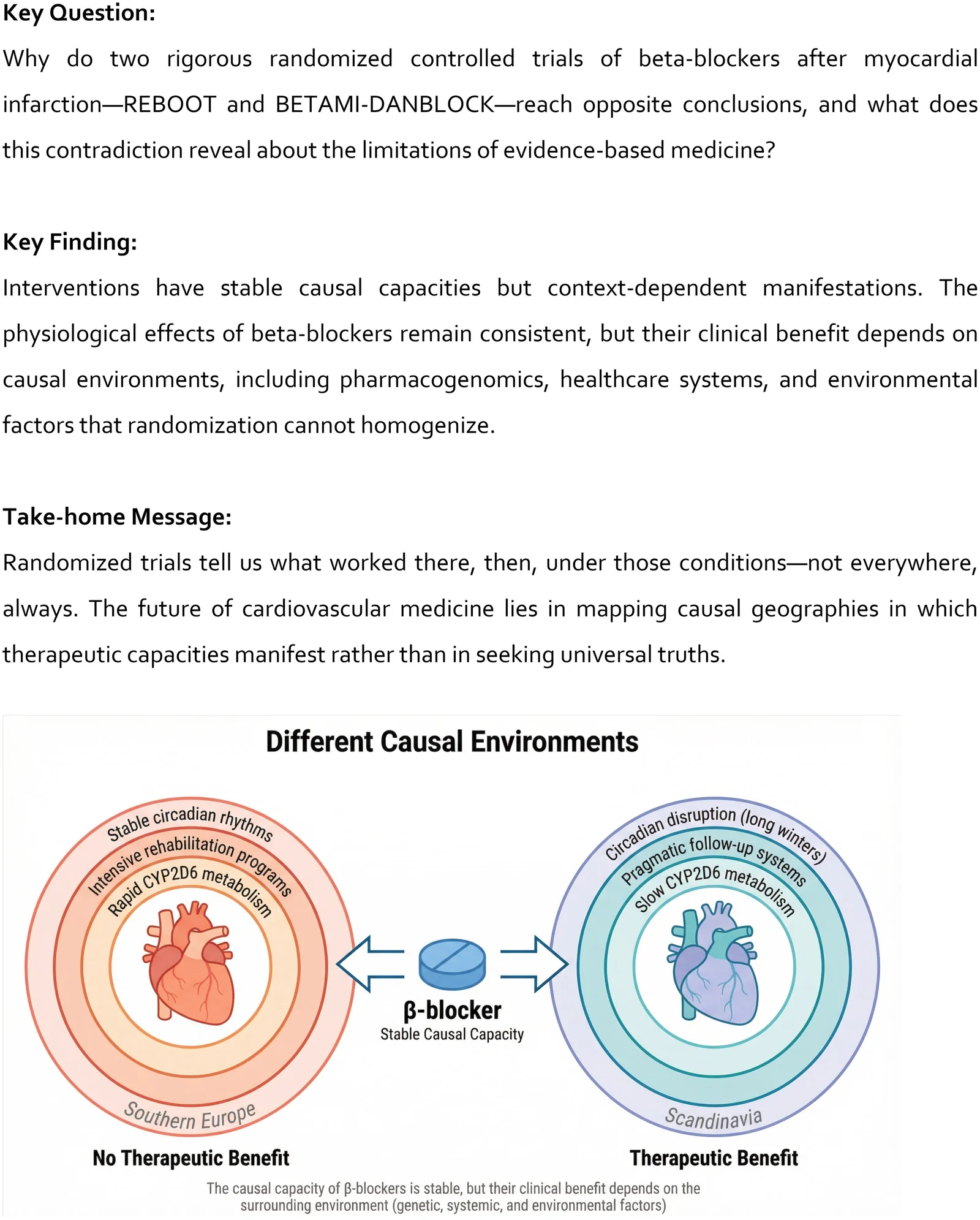

## The puzzle of divergent trials

Evidence-based medicine rests on the expectation that large, well-conducted randomized controlled trials (RCTs) addressing the same clinical question will yield concordant answers. Two contemporary trials of β-blockers unsettled this expectation. REBOOT-CNIC enrolled 8505 patients in Spain and Italy and found no significant reduction in death or major cardiovascular events among post-MI patients with preserved ejection fraction (EF).^1^ In contrast, BETAMI-DANBLOCK enrolled 5574 patients in Norway and Denmark and reported a statistically significant reduction in a composite endpoint (hazard ratio [HR] 0.85; 95% confidence interval [CI] 0.75–0.98), driven primarily by fewer non-fatal myocardial infarctions; mortality alone was unchanged.^2^ The two trials also differed in two methodological respects directly relevant to the comparison. First, the composite endpoints were not identical: BETAMI-DANBLOCK used a broader MACCE-type composite (death, myocardial infarction [MI], unplanned revascularization, ischaemic stroke, heart failure hospitalization, and malignant ventricular arrhythmia), whereas REBOOT used a narrower MACE composite (death, MI, heart failure hospitalization) that excluded cerebrovascular and arrhythmic events. Second, absolute event rates were substantially higher in the Scandinavian cohort than in the Mediterranean cohort—consistent with the well-documented north–south gradient in cardiovascular incidence across Europe—which materially increased the statistical power of BETAMI-DANBLOCK to detect a modest effect. These structural differences are themselves part of the causal environment within which each trial was conducted and cannot be neutralized by averaging. Both trials were contemporary, rigorously conducted, and embedded in modern secondary-prevention practice. Their confidence intervals overlap, suggesting quantitative rather than qualitative differences. Yet the interpretive consequences have been substantial. One trial has been labelled ‘neutral,’ while the other has been labelled ‘positive,’ which has prompted uncertainty in clinical practice. When two gold-standard RCTs diverge, the reflex is to search for a single explanatory variable that restores coherence.

## The false comfort of reductionism

The dominant explanatory move has been to canonize ejection fraction. A recent individual-patient-data meta-analysis suggested a consistent benefit of β-blockers in patients with mildly reduced EF (40–49%) and has rapidly become the prevailing interpretive lens.^3^ This response exemplifies what Cartwright has termed the false comfort of reductionism^4,5^: the tendency to explain complex causal phenomena by isolating one decisive variable. In the BETAMI-DANBLOCK trial, the overall trial was positive despite 85% of participants having preserved EF ≥50%.^2^ By contrast, the EF 40–49% subgroup—now elevated as the key therapeutic target—did not itself reach statistical significance (HR 0.82; 95% CI 0.65–1.02). Restricting β-blocker therapy to this subgroup would therefore exclude the majority of patients who contributed to the observed benefit, based on weaker evidence than the finding itself, and would explain it away. Reductionism restores analytic order but at a cost. It transforms a context-dependent signal into a rigid rule, risking converting demonstrated benefit into therapeutic omission.

The same tension is illustrated by the sex-specific findings in REBOOT, in which β-blocker therapy was associated with a statistically significant harmful effect in women^6^—a signal that could not be reproduced in BETAMI-DANBLOCK and has therefore been attributed to chance. Within our framework, this interpretation is defensible, but not the only defensible interpretation. Sex represents a biologically plausible modifier of β-blocker response: differences in heart rate, autonomic tone, and pharmacokinetics between men and women are well established, and sex-specific cardiovascular effects of adrenergic blockade have been reported in other contexts. The REBOOT sex finding exemplifies the irreducible interpretive challenge: prespecified subgroup analyses that produce isolated significant results warrant great caution, yet blanket dismissal may suppress a signal with genuine biological meaning. This is precisely the epistemic predicament that causal environment reasoning is designed to navigate—not by resolving the uncertainty, but by providing a framework for generating testable hypotheses about its source.

## Causal capacities, causal environments, and transportability

**Figure 1:**
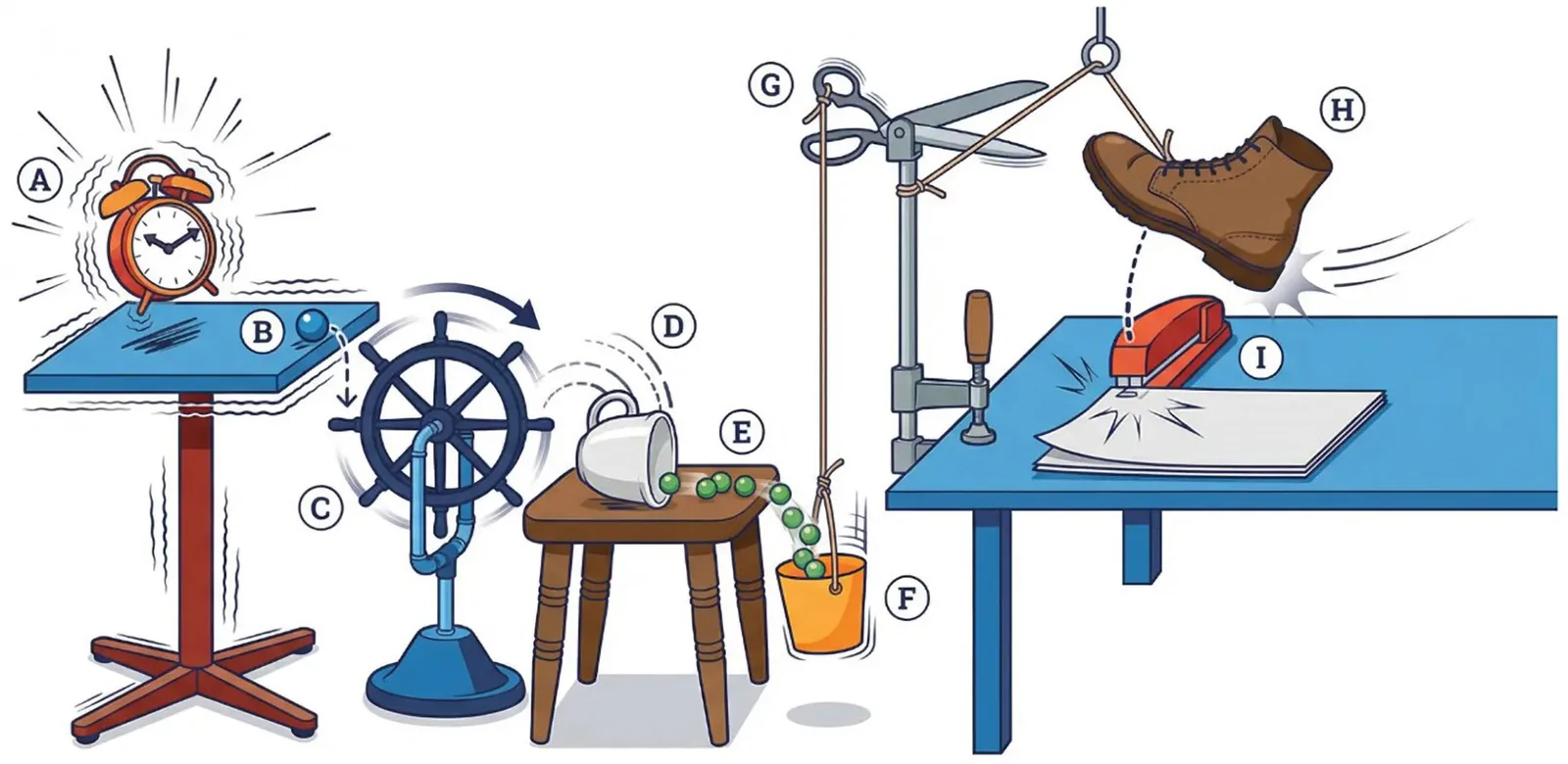
An illustration of a deterministic causal chain. This Rube Goldberg machine illustrates Cartwright's concept of a causal chain. The outcome (*I*, a stapler activated) is realized only when each intermediate step (*A–H*) is intact; failure at any single step breaks the pathway. The successful operation of the whole system constitutes a ‘causal environment’ in which the capacity of the initial event to produce the final effect is expressed. The analogy underscores that an intervention’s effects are not universal but contingent on a supportive causal structure. Modified and redrawn from an illustration of a Rube Goldberg machine published at https://kolaszek.medium.com/rube-goldberg-machine-calculations-381db05667ef.

Cartwright offers an alternative. Interventions, she argues, have causal capacities—stable potentials, such as β-blockers’ ability to reduce sympathetic drive, lower oxygen demand, suppress arrhythmia, and limit remodelling.^4,5^ But whether those capacities manifest depends on the causal environment that surrounds them. Pearl’s framework for transportability extends this insight: trial findings are valid within their source population but may not transfer to target populations with different causal structures.^7^ Randomization secures internal validity within a trial, ensuring fairness in comparing groups. It cannot, however, homogenize the conditions across populations, healthcare systems, and geographies that determine whether a drug’s capacity is expressed. The concept is intuitive through analogy. A seed has the capacity to become a tree, but only if planted in fertile soil. Antibiotics can cure infections, but only if bacteria are susceptible. So too with β-blockers: their physiological effects are stable, but their clinical manifestations depend on the scaffolding in which they are embedded. RCTs tell us what happened there, then, under those conditions. They do not guarantee what will happen everywhere, at all times (*Figure 1*).

## Causal environments in practice

While we cannot definitively establish which causal environments drove the divergence between REBOOT and BETAMI-DANBLOCK, several plausible mechanisms illustrate how Cartwright's framework could explain such discordance. A critical methodological caveat is required. The contextual mechanisms described below—pharmacogenomics, cultures of care, and climate—are *post hoc*, hypothesis-generating propositions. They are offered not as confirmatory explanations but as biologically plausible candidates for prospective testing. In the absence of prespecified analysis plans, they cannot be assigned confirmatory weight and are subject to the same risks of type I error, spurious inference, and multiplicity as any data-driven subgroup exploration. The distinction between hypothesis-generating and hypothesis-confirming evidence is not merely semantic: it determines whether a proposed mechanism warrants clinical action or further study. Our argument is that these mechanisms warrant the latter—rigorous prospective evaluation—not the former.

Pharmacogenomics: BETAMI-DANBLOCK used primarily metoprolol, metabolized by CYP2D6. Loss-of-function alleles that increase drug exposure are relatively common in Scandinavian populations, while ultra-rapid-metabolism variants that reduce exposure are more prevalent in Southern Europe.^8–10^ If these genetic differences translate into clinically meaningful differences in exposure, identical doses could yield higher circulating levels—and potentially greater clinical effect—in Norway than in Spain. Whether CYP2D6 polymorphisms actually explain trial divergence remains speculative, but the principle is sound: pharmacogenetic context can modulate drug efficacy.

### Pharmacological agent

The trials also differed at the level of the intervention itself. BETAMI-DANBLOCK used primarily metoprolol, REBOOT used bisoprolol—agents that differ in β1-selectivity, lipophilicity, and CYP2D6 dependence. Such intervention-level heterogeneity is not corrected by randomization, which allocates patients but not agents, and is a concrete source of between-trial divergence.

### Cultures of care

Healthcare delivery differs substantially across Europe. Italy and Spain often provide both inpatient and outpatient cardiac rehabilitation with structured, nurse-led follow-up. Scandinavian countries rely more on outpatient models with less intensive monitoring.^11,12^ It is plausible that in REBOOT, intensive background care already captured much of the remaining potential benefit from optimal secondary prevention, leaving little incremental effect for β-blockers to deliver. In BETAMI, where follow-up was less structured, β-blockers may have compensated for gaps in background management. This hypothesis—that drugs compete with and complement systems of care—remains untested but illustrates how system-level factors could render an intervention vital in one context yet superfluous in another.

### Climate and circadian stressors

Long Nordic winters disrupt circadian rhythms and are associated with heightened sympathetic tone and increased arrhythmic risk. Whether this environmental stressor amplifies the benefit of β-blockers remains speculative, but the biological plausibility is clear: sympatholytic therapy may prove more protective in proarrhythmic environments. In Mediterranean environments, where sympathetic activation is lower, incremental benefit may be correspondingly muted.

This pattern—where treatment effectiveness depends on causal environment rather than mechanism alone—transcends cardiology. Precision oncology has revealed that molecularly targeted therapies succeed only when the genomic and microenvironmental context permits.^13^ These examples, while speculative in their specific application to β-blocker trials, demonstrate that REBOOT and BETAMI-DANBLOCK are not mutually exclusive. Both can be right, each within its causal environment. The aim is not to prove which environments mattered, but to show that Cartwright’s framework offers a coherent alternative to reductionist explanations.

The framework does not license the unlimited proliferation of unmeasured modifiers. Candidate modifiers must be biologically plausible, prespecifiable, and testable—criteria the hypotheses advanced here satisfy and that registry-based phase-4 trials are designed to evaluate.

## Why meta-analysis cannot deliver universality

When randomized trials diverge, meta-analysis is often invoked as the final arbiter. Two highly influential meta-analyses addressed β-blockers post-MI: the Lancet analysis of mildly reduced EF (40–49%) reporting consistent benefit^3^ and Kristensen et al.’s synthesis of preserved EF (≥50%) finding no overall benefit.^14^ Both are methodologically sophisticated, and their conclusions warrant serious attention. Yet pooling across trials conducted in distinct contexts does not generate universality; it produces an average. This distinction matters. An average treatment effect may be statistically coherent while concealing systematic variation driven by differences in causal environments.

Meta-analyses assume treatment effects transport across populations, but Pearl and Bareinboim’s transportability framework shows this requires specific conditions.^7^ When populations differ in unmeasured factors that modify treatment effects—such as genetic variants, healthcare system intensity, or environmental stressors—averaging produces a summary that may not apply to any specific context. The discrepancy between REBOOT and BETAMI may reflect differences in these unmeasured causal structures rather than failure of the intervention itself. Current meta-analytic frameworks cannot assess whether effects vary by unmeasured contextual factors, only by measured patient characteristics like ejection fraction. Estimating context-specific effects would require understanding which aspects of the causal structure differ across settings—precisely what Pearl’s transportability analysis addresses, but current meta-analyses ignore.

Adherence to therapy represents one such unmeasured factor. Compliance is not merely individual behaviour but a property of healthcare systems, follow-up intensity, patient education, cultural norms, and tolerability. Even modest differences in long-term adherence can materially alter observed effects for drugs whose benefits accrue over time. Adherence is rarely harmonized across trials. Meta-analytic models implicitly assume comparable exposure when effective doses may differ substantially. An individual-patient-data meta-analysis cannot fully overcome this limitation—system-level adherence patterns are rarely captured. These unmeasured differences may masquerade as biological effect modification or be misattributed to ejection fraction. The resulting coherence is statistical rather than mechanistic.

## When should negative results neutralize positive evidence?

The situation becomes particularly vexing when meta-analytic averages conflict with well-established physiological mechanisms. Several principles warrant consideration. Negative results merit greater weight when they arise from large, well-conducted trials in populations that closely resemble clinical practice. Physiological mechanisms retain relevance when they have been directly demonstrated in humans and when alternative explanations—such as differences in adherence, co-interventions, or environmental stressors—remain plausible. Finally, the magnitude of observed effects matters: modest, inconsistent benefits that hover near the threshold of statistical significance demand greater scrutiny. In the case of β-blockers post-MI with preserved ejection fraction, the effect, if present, appears limited and conditional—a signal that cautions against universal application but does not justify blanket dismissal in all contexts.

Seen in this light, contemporary meta-analyses do not contradict the divergent findings of REBOOT and BETAMI-DANBLOCK; rather, they smooth them out. The risk is that this smoothing is mistaken for explanation. By canonizing EF as the organizing principle, meta-analysis may inadvertently reify a surrogate marker for a constellation of unmeasured causal environments.

The meta-analyses cited encompass five trials—REBOOT, BETAMI-DANBLOCK, REDUCE-AMI^15^ (Sweden), and CAPITAL^16^ (Japan)—and report low statistical heterogeneity. We acknowledge that low *I*^2^ formally justifies pooling under standard meta-analytic assumptions, and that the pooled estimates represent the best available summary of the trial evidence. However, several cautions apply. First, heterogeneity tests have severely limited statistical power when the number of included trials is small. With only five trials, Cochran *Q* and *I*^2^ are unlikely to detect true contextual heterogeneity even when it is substantial; a low *I*^2^ in this setting is therefore better interpreted as ‘heterogeneity not detected’ than ‘heterogeneity absent.’ Second, Pearl and Bareinboim’s transportability framework reveals that statistical homogeneity across trial settings does not guarantee transportability to a given clinical context. Five trials can yield similar average effects while each averages over different underlying distributions of contextual modifiers, so the pooled estimate represents an average of averages—statistically coherent but potentially misleading for any specific patient in any specific clinical setting. This concern is heightened by the inclusion of CAPITAL, conducted in Japan, a setting with well-documented differences in CYP2D6 allele frequencies, healthcare structure, and lifestyle factors relative to both Scandinavia and Southern Europe. Third, neither of these meta-analyses conducted a formal region-by-treatment interaction test, which would be the most direct empirical test of the hypothesis we advance. These considerations align directly with the GRADE framework for rating the certainty of evidence, which formally rates down for inconsistency when point estimates or directions of effect vary across populations and settings, and for imprecision when confidence intervals span clinically distinct decisions.^17–20^ Crucially, GRADE explicitly recognizes that statistical homogeneity does not equate to clinical or contextual homogeneity, and that a small number of trials limits the credibility of any judgment of consistency.^18,20^ Conventional meta-analytic pooling therefore does not exempt the evidence from these qualitative judgments; if anything, transportability concerns strengthen the case for downgrading certainty when contextual modifiers cannot be ruled out, and for presenting context-specific rather than universally framed recommendations.

## Clinical and guideline implications

The lesson is not that one trial was right and the other wrong. Both were correct within their respective contexts. The danger lies in over-interpretation. If guidelines restrict β-blocker therapy exclusively to patients with EF 40–49%, many patients with preserved EF may be denied a therapy that could benefit them in supportive environments. Guideline evolution should therefore proceed cautiously.

Registry-based, cluster-randomized phase-4 trials offer a framework for learning while doing. The SWITCH-SWEDEHEART study exemplifies this approach: a stepped-wedge design embedded within the Swedish national quality registry that tests treatment policies under real-world conditions while preserving randomized oversight.^21^ Such designs allow policies to be tested in actual healthcare environments, capturing the full complexity of adherence, co-interventions, and system-level factors that shape treatment manifestation. Phase-4 evaluation becomes particularly critical when phase-3 trials—often conducted under idealized conditions or in selected populations—produce promising but contextually limited results. Registry-based trials leverage cohesive healthcare systems to generate rigorous, ecologically valid evidence.

The causal environment framework is not a counsel of therapeutic nihilism. Its practical implication is a research agenda: prospective registry-based trials should be designed to stratify by pharmacogenomic profiles, catalogue cardiac rehabilitation intensity, and capture environmental stressors as covariates—not as *post hoc* subgroup explorers, but as prespecified causal environmental variables. Future β-blocker trials in preserved EF post-MI should incorporate CYP2D6 genotyping, standardized rehabilitation intensity scoring, and geographic and seasonal co-variables as prespecified effect modifiers.

## Conclusion: toward precision causality

Randomized trials remain the cornerstone of clinical evidence. But they do not deliver universal decrees. They describe what worked under specific conditions. The divergence between REBOOT and BETAMI-DANBLOCK exposes the limits of assuming that randomization alone guarantees generalizability. Precision medicine is not only genomic; it is contextual. β-blockers after MI are neither universally beneficial nor universally futile—they are conditionally effective. The benefit, if present, appears modest and highly dependent on the causal environment. The future of cardiovascular medicine lies not in reducing complexity to a single variable, but in mapping the causal environments in which therapeutic capacities become manifest.

## Data Availability

This manuscript did not generate, analyse, or report original datasets. It is based on the authors’ interpretation of published studies and conceptual literature.
